# From Antimicrobial Biomaterials to Infection-Resilient Interfaces: Engineering the Host, Microbe, and Material Interface Against Drug-Resistant Biofilms

**DOI:** 10.3390/life16101670

**Published:** 2026-10-07

**Authors:** Ayman Elbehiry, Adil Abalkhail, Eman Marzouk, Abdulraheem Bayameen, Shaimaa Ashour, Faisal Yousuf, Fahad Albugami, Sulaiman Anagreyyah, Abdulaziz M. Almuzaini, Mai Ibrahem, Husam M. Edrees

**Affiliations:** 1 Department of Public Health, College of Applied Medical Sciences, Qassim University, P.O. Box 6666, Buraydah 51452, Saudi Arabiae.marzouk@qu.edu.sa (E.M.); 2 Family Medicine Department, King Fahad Armed Forces Hospital, Jeddah 23311, Saudi Arabia; 3 Human Resources Department, Prince Mansour Military Hospital, Taif 26526, Saudi Arabia; 4 Department of Veterinary Preventive Medicine, College of Veterinary Medicine, Qassim University, Buraydah 51452, Saudi Arabia; 5 Department of Public Health, College of Applied Medical Science, King Khalid University, Abha 61421, Saudi Arabia; 6 Department of Physiology, Faculty of Medicine, University of Tabuk, Tabuk 71491, Saudi Arabia; hedrees@ut.edu.sa

**Keywords:** antimicrobial biomaterials, antibiotic resistance, drug-resistant biofilms, biomaterial-associated infection, infection-resilient interfaces, biofilm control, host-microbe-material interactions, antimicrobial surfaces, medical devices

## Abstract

Drug-resistant biofilm infections remain a major challenge for medical devices and implants. Biofilm formation can reduce antimicrobial effectiveness through physiological heterogeneity, tolerance, persistence, and interactions with the host and biomaterial surface. Antimicrobial biomaterials have been developed to prevent colonization, detect infection, provide antimicrobial activity, control established biofilms, and support tissue recovery. However, their performance depends on the biological context in which they function. This review examines these strategies through interactions among the host, microbe, and material, using carbapenem-resistant *Acinetobacter baumannii*, methicillin-resistant *Staphylococcus aureus*, and carbapenem-resistant *Pseudomonas aeruginosa* as selected biological stress-test examples for evaluating biomaterial performance. It also considers the limitations in current evaluation, including planktonic testing, biological conditioning, microbial adaptation, effects on non-target microorganisms, material stability, manufacturing, and clinical translation. Antimicrobial activity alone does not establish effective performance in infection. Material function must match the microbial state, host environment, and intended clinical use while maintaining host compatibility and function during use. We propose infection resilience as a design framework organized around Prevent, Detect, Respond, Resolve, and Regenerate. Infection resilience refers to coordinated material performance under changing microbial and host conditions rather than the number of functions incorporated into a material. This framework offers a practical basis for more biologically relevant evaluation and purposeful design of antimicrobial biomaterials for drug-resistant biofilm infections.

## 1. Introduction

Antimicrobial resistance (AMR) is an important global health challenge that limits the effectiveness of antibacterial treatment and creates a continuing need for improved strategies to prevent and manage bacterial infections. In the 2024 World Health Organization (WHO) Bacterial Priority Pathogens List, carbapenem-resistant *Acinetobacter baumannii* (CRAB) is classified as a critical-priority pathogen, whereas carbapenem-resistant *Pseudomonas aeruginosa* (CRPA) and methicillin-resistant *Staphylococcus aureus* (MRSA) are classified as high-priority pathogens. The list was developed to guide research and development and inform strategies to prevent and control AMR [1].

The problem is particularly important for infections associated with medical devices and implants. Bacteria can rapidly adhere to biomaterial surfaces and form biofilms that complicate antimicrobial treatment and host defense, making implant-associated infections difficult to eradicate [2,3]. This review focuses on bacterial biofilm-associated infections involving antimicrobial biomaterials, with particular emphasis on medical devices and implants. Fungal biofilms and other non-bacterial microbial infections are outside the primary scope of the review. Accordingly, the term “microbial” is retained only where a broader biological context is intended; the evidence and examples discussed throughout the review are predominantly bacterial. A limited food-industry example, *Listeria monocytogenes* (*L. monocytogenes*), is included in Part 4 because biofilm control on food-contact surfaces provides a distinct application context for antimicrobial biomaterials. This example does not change the review’s primary focus on medical devices and implants.

Biofilm-associated infections cannot be understood solely through the conventional framework of AMR. Bacteria within biofilms occupy diverse physiological states and experience spatial variation in oxygen, nutrients, metabolic activity, and other local conditions that distinguish biofilm populations from planktonic cells [4,5]. These conditions can increase tolerance to antimicrobial treatment without requiring the genetic resistance mechanisms that define conventional AMR [5]. Persister cells add another layer of complexity because these phenotypic variants can enter dormant or slow-growing states and survive otherwise lethal antimicrobial exposure without necessarily acquiring inherited resistance mechanisms [6]. Their survival can contribute to persistence and complicate the eradication of chronic infections [5,6].

These challenges have stimulated extensive development of antimicrobial biomaterials. Current approaches include antiadhesive and antifouling surfaces, contact active materials, antimicrobial releasing systems, surface modified biomaterials, and multifunctional platforms designed to prevent bacterial attachment, inhibit biofilm formation, or act against established biofilms [7,8,9].

However, increasing antimicrobial potency alone does not resolve the translational challenges of biomaterial-associated infection. Performance depends on the microbial state being tested and on whether the material is intended to prevent colonization or control established infection [3,10]. In a study of clinical staphylococcal isolates from implant-associated infections, most isolates formed biofilms, and the minimum biofilm eradication concentrations were substantially higher than the corresponding minimum inhibitory concentrations (MICs) [10]. Recent reviews also identify biocompatibility, cytotoxicity, long-term stability, scalability, regulatory requirements, and limitations of experimental models as important challenges for translating antimicrobial biomaterials into clinical applications [7,11].

An additional complication is that a biomaterial surface is rapidly modified after exposure to biological fluids. After implantation, proteins from blood and tissue fluids rapidly adsorb to the biomaterial and form a conditioned interfacial layer whose composition and conformation depend on both the material surface and the surrounding biological environment [12]. This conditioned interface can therefore differ from the original material surface and alter subsequent interactions with microorganisms and host cells [12]. Recent experimental work illustrates this context dependence. In a polydimethylsiloxane model, serum albumin preadsorption reduced initial attachment of *Staphylococcus aureus* (*S. aureus*) and *Staphylococcus epidermidis* (*S. epidermidis*), whereas prolonged exposure to albumin conditioned surfaces promoted *P. aeruginosa* attachment and biofilm formation under prolonged exposure and was associated with altered bacterial physiological responses and reduced macrophage-mediated bacterial clearance [13].

The biomaterial tissue interface also influences host responses to both the material and invading microorganisms. In implant-associated infections, bacterial adhesion, and biofilm formation occur alongside host immune responses, while implant-infecting bacteria can evade innate and adaptive host defenses [2]. This has encouraged interest in host-directed strategies that complement direct antibacterial activity by supporting protective immune functions, particularly those of neutrophils and macrophages [14].

These observations point to a broader problem in how antimicrobial biomaterials are evaluated. Antimicrobial activity measured under controlled laboratory conditions does not necessarily predict performance against established biofilms in biologically complex environments, where microbial tolerance, surface conditioning, and host responses can influence treatment outcome [5,10,13]. Thus, antimicrobial biomaterials should be evaluated in relation to the biological context in which they are expected to function rather than by bactericidal activity alone.

These considerations support a shift from viewing antimicrobial biomaterials primarily as bacteria-killing materials toward designing interfaces that remain effective within the biological complexity of infection. In this review, we use the term infection-resilient interface to describe a biomaterial system designed to limit pathogen colonization, respond appropriately to infection, control persistent biofilm populations, remain compatible with host defense, and support tissue recovery. This terminology is used here as an organizing framework rather than as an established classification of biomaterials. It builds on established approaches including infection-resistant and antiadhesive surfaces, as well as multifunctional antimicrobial biomaterials [15,16]. The contribution proposed here is the integration of established functions across changing infection stages and biological conditions. The broader rationale is consistent with recent efforts to understand implant-associated infection as a dynamic interaction among host biology, microbiota, biomaterial properties, and environmental factors [17].

Building on this perspective, we organize the review around five biological objectives: Prevent, Detect, Respond, Resolve, and Regenerate. Prevention addresses microbial attachment and colonization before a mature biofilm develops. Detection and response encompass recognition of infection-associated conditions and activation of appropriate antimicrobial functions, whereas resolution concerns established biofilms and persistent bacterial populations. Regeneration refers to restoration of a functional host material interface after infection control. These objectives provide the organizing framework for the review.

Drug-resistant pathogens provide a stringent context in which to examine the performance of antimicrobial biomaterials. In this review, CRAB, CRPA, and MRSA are used as selected biological stress-test examples rather than as representative organisms for all drug-resistant or biomaterial-associated infections [1,18]. They provide contrasting Gram-negative and Gram-positive challenges involving resistance, surface-associated growth, persistence, and adaptation. Other bacteria are relevant to biomaterial-associated infections, including coagulase-negative staphylococci such as *S. epidermidis*, which are well established in device-associated biofilm infections [19]. Its established association with device-related biofilm infections provides a separate clinical rationale for considering it when evaluating antimicrobial biomaterials [19]. Thus, CRAB, CRPA, and MRSA are used here to examine complementary resistance-focused biological challenges, not to provide a comprehensive representation of the pathogens involved in biomaterial-associated infections.

Accordingly, this review is not organized around a material composition or fabrication method. Instead, it examines antimicrobial biomaterials through the biological challenges they must overcome across the course of infection, integrating bacterial ecology, biofilm physiology, host defense, microbial adaptation, and translational constraints [5,11,17]. The central premise is that successful antimicrobial biomaterials should be evaluated not only by antimicrobial activity, but also by their ability to maintain infection control while preserving a functional host-material interface. This perspective is particularly relevant to biomaterials that seek to combine infection control with immune compatibility and tissue recovery [14,17].

## 2. Literature Search Strategy

This narrative review was developed through a focused literature search to identify studies relevant to antimicrobial biomaterials, drug-resistant biofilms, biomaterial-associated infection, host responses, microbial adaptation, and infection control at the host, microbe, and material interface. Searches were conducted using PubMed, Scopus, Web of Science, and Google Scholar. The final literature search was conducted on 26 September 2026. Search terms included combinations of “antimicrobial biomaterials,” “antimicrobial surfaces,” “drug-resistant biofilms,” “biofilm-associated infection,” “biomaterial-associated infection,” “biofilm tolerance,” “persistence,” “antibiotic resistance,” “antibiofilm materials,” “responsive biomaterials,” “immunomodulatory biomaterials,” “host-microbe interactions,” and “tissue recovery.” Representative search strings combined these terms using the Boolean operators AND and OR. For example, searches included (“antimicrobial biomaterials” OR “antimicrobial surfaces”) AND (biofilm OR “biofilm-associated infection”) and (“biomaterial-associated infection” OR “implant-associated infection”) AND (biofilm OR bacteria).

The search emphasized the literature published from 2004 to 2026, with consideration of earlier studies when necessary to provide relevant biological or conceptual context. The 2004 starting point was selected to focus the review on more recent developments in antimicrobial biomaterials and biofilm research, while earlier studies were retained when they provided important foundational biological or conceptual evidence. Publications were considered based on their relevance to the major themes addressed in the review, including prevention of microbial attachment, antimicrobial and antibiofilm strategies, responsive materials, control of established biofilms, host compatibility, microbial resistance and tolerance, biological realism of experimental models, material durability, and translational feasibility. Particular attention was given to studies evaluating antimicrobial biomaterials against clinically relevant or drug-resistant pathogens and to research addressing differences between planktonic bacteria and established biofilms.

Additional relevant publications were identified by screening the reference lists of key articles and reviews. Studies were not considered when they fell outside the scope of antimicrobial biomaterials or biofilm-associated infection and did not provide information relevant to the biological, material, or translational themes of the review. Original research articles were prioritized for experimental findings and material performance, whereas review articles were mainly used for background, established concepts, and identification of additional relevant studies. The search used English-language terms, and no additional language restriction was applied during study consideration. Because this is a narrative review, no formal systematic-review protocol, risk-of-bias assessment, or quantitative meta-analysis was performed. Instead, evidence from biomaterials science, microbiology, infection biology, immunology, and translational research was critically synthesized to develop an integrated perspective on biomaterial performance under clinically relevant infection conditions.

The literature was not organized solely according to material composition. Instead, studies were considered in relation to the biological challenges that antimicrobial biomaterials must address across infection, including microbial attachment and biofilm development, antimicrobial response, host compatibility, microbial adaptation, persistence, and tissue recovery. This approach supports the central premise of the review that antimicrobial activity alone is insufficient to define biomaterial performance and that material function must be considered within the changing host, microbe, and material interface. A total of 131 sources were included in the final review. Because this was a narrative review and no formal screening log was maintained, the exact number of records identified and screened across the databases was not prospectively recorded.

## 3. The Host, Microbe, and Material Interface: Understanding Where Infection Begins

An implanted or applied biomaterial interacts with microorganisms within a biological environment shaped by both the material and the host. Its biological performance emerges from interactions among material properties, microbial behavior, and host responses, with each component influencing the environment experienced by the others [20,21]. The surface encountered by microorganisms in vivo is therefore not necessarily identical to the surface characterized before implantation. Protein adsorption, immune cell recruitment, fluid exposure, mechanical forces, and material degradation can progressively alter the interface and its biological function [12,22].

### 3.1. The Material Interface

Material properties shape the earliest biological interactions after implantation or application. Surface chemistry, charge, wettability, surface roughness and topography, mechanical properties, and degradation can affect protein adsorption, bacterial attachment, immune cell behavior, and tissue integration. These variables are often interdependent rather than independent determinants of antimicrobial performance. For example, changing surface chemistry may simultaneously alter surface charge, wettability, protein adsorption, and subsequent biological conditioning. Therefore, an antimicrobial effect should not be attributed to a single surface parameter unless the experimental design adequately isolates that variable [9,12].

Surface chemistry determines the chemical groups available at the interface and affects surface energy, charge, protein adsorption, and interactions with microorganisms and host cells. On titanium-based materials, differences in surface characteristics can alter the adsorption and organization of proteins, providing an important link between material chemistry and subsequent biological responses [12]. Surface charge contributes to electrostatic interactions between bacterial cells and biomaterial surfaces. Because many bacterial cell envelopes carry a net negative surface charge under physiological conditions, changes in material charge can modify bacterial attachment. However, the magnitude and direction of this effect depend on bacterial species, ionic conditions, surface chemistry, and the presence of adsorbed biological molecules; surface charge should not be regarded as an isolated predictor of colonization [9,23].

Wettability influences hydration and interfacial interactions and can affect both protein adsorption and bacterial attachment. However, wettability does not provide a universal predictor of biological performance. Studies of clinically relevant titanium surfaces indicate that macrophage responses depend on hydrophilicity together with other surface characteristics and biological factors [9,12]. Surface roughness and topography provide physical cues that can influence bacterial adhesion as well as host–cell attachment and organization. Micro- and nanoscale features can change the effective contact between microorganisms and the surface, but their effects are strongly dependent on feature dimensions, bacterial characteristics, surface chemistry, and the surrounding environment. Consequently, roughness should not be classified simply as either antibacterial or bacteria promoting; the biological outcome depends on the specific topographical design [24,25].

Mechanical properties add another layer to the interface. Stiffness, deformation, and other physical cues can influence macrophage behavior and inflammatory signaling [20,26]. This makes mechanical design relevant not only to structural performance but also to the biological response surrounding an implant.

Degradation introduces an explicitly time-dependent component. During degradation, changes in material properties and degradation products can alter the cellular environment and material function. Thus, the biological performance of a degradable biomaterial cannot be inferred solely from its initial properties because those properties may change substantially during use [22]. The important implication is that material properties should not be evaluated solely according to their direct effects on microorganisms. The same surface characteristics that influence bacterial attachment can also alter protein adsorption, immune cell behavior, inflammation, and tissue integration [27,28].

### 3.2. The Microbial Interface

For microorganisms, the transition from free-living cells to surface-associated communities represents a major change in biological state. Biofilm development generally involves initial attachment, stabilization of surface-associated cells, microcolony development, matrix production, maturation, and eventual dispersal, although the sequence and underlying mechanisms vary among bacterial species and environmental conditions [29,30]. Initial contact and adhesion are governed by physicochemical and biological interactions between the bacterial cell, the material surface, and any conditioning layer present at the interface. Long-range interactions can bring cells into proximity, followed by shorter range interactions that may stabilize attachment through adhesins, surface appendages, and other bacterial structures [29,31].

Once stable attachment is established, bacterial growth and extracellular matrix production support the development of microcolonies and a structured biofilm. The extracellular polymeric substance (EPS) matrix is composed of polysaccharides, proteins, nucleic acids, lipids, and other biomolecules and provides structural cohesion while influencing adhesion, transport, and the local chemical environment of the community [30,32]. However, the EPS matrix should not be considered a universal physical barrier to antimicrobial penetration. Its effects depend on the antimicrobial agent, matrix composition, and local biofilm conditions [5].

Biofilm maturation creates a spatially heterogeneous microbial environment rather than a uniform population. Differences in oxygen, nutrients, metabolic activity, waste products, and signaling molecules generate microscale physiological gradients and distinct bacterial states within the same biofilm [4]. These differences contribute to antimicrobial tolerance and help explain why established biofilms can be substantially more difficult to eradicate than planktonic populations [5]. Reduced growth rate, metabolic heterogeneity, stress responses, oxygen and nutrient limitation, altered gene expression, and persister populations can all contribute to antimicrobial tolerance [5,6]. Phenotypic adaptation further increases this heterogeneity. Biofilm bacteria can alter gene expression, metabolism, stress responses, surface properties, and other cellular functions as local conditions change. In *S. aureus*, biofilm development involves coordinated developmental changes that produce physiologically distinct cellular states within the community [33].

Persistence and tolerance represent important consequences of this physiological organization. Slow-growing or dormant cells can survive antimicrobial exposure without relying on conventional resistance mechanisms, while the biofilm matrix and heterogeneous microenvironment can further reduce antimicrobial effectiveness [5,6]. These physiological forms of tolerance should be distinguished from genetic AMR, which involves heritable changes that reduce susceptibility to an antimicrobial agent [5]. Thus, susceptibility genotype alone does not adequately describe the microbial state at a biomaterial interface. Its behavior also depends on physiological state, spatial organization, metabolic activity, and local environmental conditions.

### 3.3. The Host Interface

After implantation, proteins from blood and tissue fluids rapidly adsorb to the biomaterial and form a conditioned interfacial layer whose composition and conformation depend on both the material surface and the surrounding biological environment [12,34].

This protein layer provides an important connection between material properties and host recognition. Surface chemistry, topography, charge, and wettability can influence protein adsorption, while the adsorbed proteins can subsequently alter cellular adhesion, signaling, and immune recognition [12,28]. The host therefore responds to a biologically conditioned interface rather than necessarily to the original material surface.

Innate immune recognition is an early component of the host response to implanted biomaterials. Implantation produces tissue injury and activates inflammatory pathways, while adsorbed proteins and complement components provide additional signals that can recruit and activate immune cells [14,21].

Neutrophils are important early effector cells in this response. They contribute to microbial control through phagocytosis, reactive oxygen species (ROS) production, degranulation, and neutrophil extracellular trap formation. Their activity can promote bacterial clearance, but excessive or prolonged activation can also contribute to tissue injury and persistent inflammation [14]. The relevant objective is therefore an effective neutrophil response that supports microbial control while limiting tissue injury and persistent inflammation.

Macrophages represent a second major cellular component of the biomaterial interface. They respond to material-derived physical and chemical cues and participate in phagocytosis, inflammatory regulation, foreign body reaction, tissue repair, and integration. Their phenotype and function can be influenced by surface topography, chemistry, stiffness, and other features of the biomaterial [26,27].

The host response is not simply divided into beneficial inflammation and harmful inflammation. An appropriately regulated inflammatory response can support both host defense and tissue repair, whereas persistent or dysregulated inflammation can promote foreign body reaction, fibrosis, and impaired tissue integration [21,28]. Thus, tissue integration represents an active biological process instead of a passive consequence of material implantation. Surface properties can influence immune cell recruitment and behavior, inflammatory signaling, and subsequent tissue organization, linking the early host response to the long-term performance of the biomaterial [27,35].

### 3.4. The Interface Is Dynamic

The material, microbial, and host components of the interface cannot be considered independently. Material properties influence microbial attachment and host–cell behavior, microorganisms alter the local environment through growth and matrix production, and host proteins and immune cells continually modify both the material surface and the microbial niche [12,20]. This interaction becomes particularly important after biological exposure because the original material surface progressively acquires new biological characteristics. Protein adsorption changes the molecular interface, immune responses alter the surrounding inflammatory environment, bacterial growth changes local nutrient and oxygen conditions, and degradation can progressively modify the material itself [12,22].

Consequently, antimicrobial performance measured on a pristine surface may not predict performance after biological conditioning. Surface modifications can alter both microbial attachment and host compatibility, emphasizing the need to evaluate the conditioned interface under relevant biological conditions. A modification that reduces bacterial attachment under simplified experimental conditions may behave differently after biological conditioning, while a modification that enhances direct antibacterial activity may also alter host compatibility or tissue integration [9,27]. Hence, where relevant to the intended application, antimicrobial biomaterials should also be tested after biological conditioning, such as preconditioning with serum proteins or other relevant biological fluids, rather than relying only on freshly prepared surfaces [12,13].

**Figure 1:**
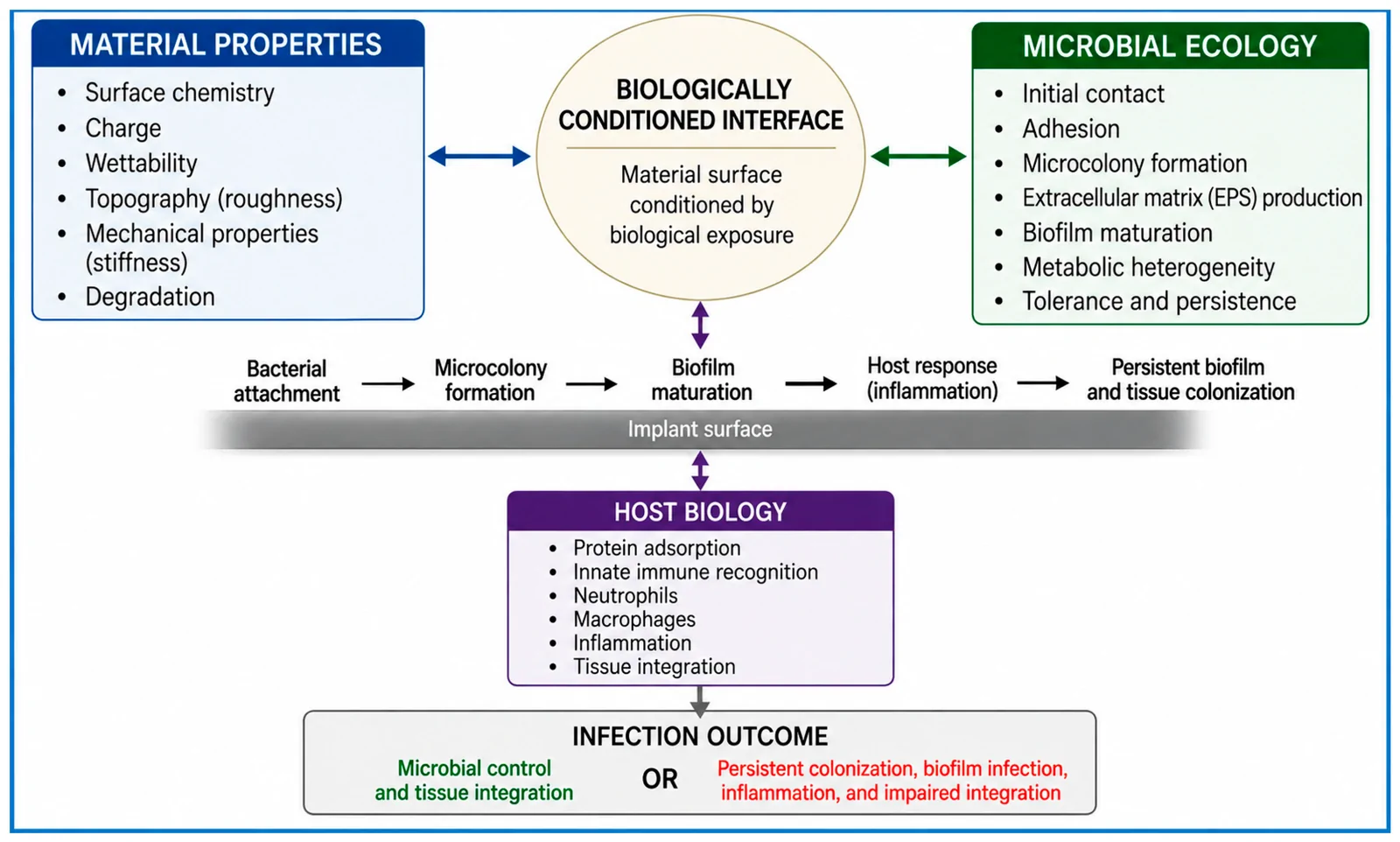
The host, microbe, and material interface. Material properties, microbial ecology, and host biology interact at a biologically conditioned implant interface. These interactions influence bacterial attachment, biofilm development, host response, tissue integration, and infection outcome.

The clinically relevant antimicrobial interface is therefore the biologically conditioned interface that develops after implantation or application, not simply the pristine surface fabricated in the laboratory. Figure 1 summarizes this dynamic interface.

## 4. Drug-Resistant Biofilms as a Stringent Test of Biomaterial Performance

Once infection is established, antimicrobial biomaterials must be evaluated against the microbial state they are intended to control. Biofilm growth changes bacterial physiology and antimicrobial susceptibility, creating a biological context that is not fully represented by conventional planktonic testing [36,37]. For antimicrobial biomaterials, the appropriate test should reflect the biological function being evaluated. Materials designed to prevent initial colonization should be assessed during early bacterial attachment, whereas materials intended to control established infection should also be evaluated against established biofilms under conditions relevant to their intended application [37].

### 4.1. Why Planktonic Susceptibility Is an Inadequate Sole Benchmark

Planktonic susceptibility testing provides a standardized measure of antimicrobial activity against freely suspended bacteria and remains essential for conventional antimicrobial susceptibility assessment. However, results obtained with planktonic cells do not necessarily predict antimicrobial activity against bacteria growing within established biofilms [36,37].

Biofilm-specific studies have demonstrated quantitative and qualitative differences between antimicrobial responses in planktonic and biofilm populations. Measures such as the minimum biofilm inhibitory concentration and minimum biofilm eradication concentration have been used to characterize antimicrobial activity against biofilm-associated bacteria, although methods and definitions vary and clinically validated breakpoints remain limited [36,37]. 

Importantly, biofilm studies can measure different outcomes that should not be treated as equivalent. These include reduction in initial adhesion, inhibition of biofilm formation, reduction in established biofilm biomass or metabolic activity, reduction in viable cells, prevention of regrowth, and eradication of an established biofilm. The appropriate endpoint depends on the intended function of the biomaterial, and biomass or metabolic reduction alone does not demonstrate eradication of viable bacteria [37,38].

This distinction is particularly relevant to biomaterials because antimicrobial activity may be assessed at different stages of microbial colonization. A surface that reduces initial bacterial attachment may not eradicate an established biofilm, whereas a material designed to deliver an antimicrobial agent must demonstrate activity against the microbial population present at the stage targeted by the material. Testing should therefore be matched to the material’s intended antimicrobial function and the microbial state it is designed to control [36,37,39].

Planktonic assays are not inadequate in themselves. They become insufficient when used as the sole evidence of performance against established surface-associated biofilms. Their appropriate role is initial antimicrobial characterization, followed by testing that reflects the microbial state and biological function targeted by the biomaterial [36,37].

### 4.2. Why Established Biofilms Create a More Stringent Biological Challenge

Established biofilms contain spatially heterogeneous bacterial populations embedded within an extracellular matrix. Differences in nutrient availability, oxygen concentration, metabolic activity, and local chemistry can generate physiological heterogeneity within the same biofilm [4,5]. This heterogeneity affects antimicrobial response. Actively growing cells may respond differently from slow-growing or dormant populations, allowing some cells to survive antimicrobial exposure without possessing genetically encoded resistance to the agent [5,40].

The extracellular matrix can also modify antimicrobial exposure through interactions that affect transport, binding, and local chemical conditions. These effects vary with matrix composition, antimicrobial properties, and biofilm structure, so the matrix should not be regarded as a universal physical barrier to antimicrobial penetration [32,41]. Biofilm age is therefore an important experimental variable. Established biofilms can display greater antimicrobial tolerance than early surface-associated populations, and differences in biofilm development and experimental conditions can substantially affect measured antimicrobial efficacy [36,41]. 

Measured biofilm susceptibility can also be influenced by the growth medium, surface material, hydrodynamic conditions, nutrient availability, inoculum, and incubation conditions [36,37,41]. Therefore, comparisons between antibiofilm studies require sufficiently detailed and, where possible, standardized experimental conditions. Persistence adds a further challenge. Persister cells are phenotypically tolerant subpopulations that can survive antimicrobial exposure without requiring inherited resistance to the antimicrobial, allowing viable cells to remain after treatment [5,40].

Genetic resistance, tolerance, and persistence should therefore be distinguished. Resistance refers to heritable characteristics that reduce antimicrobial susceptibility, whereas tolerance describes survival or slower killing during antimicrobial exposure without necessarily changing susceptibility as measured by the MIC; persistence refers to survival of a phenotypically distinct subpopulation during exposure [5]. These distinctions have direct implications for biomaterial testing. Prevention of initial attachment, inhibition of biofilm formation, reduction in established biofilm biomass, and reduction or eradication of viable biofilm cells are different endpoints and should not be presented as equivalent measures of antimicrobial performance. A rigorous study should therefore specify the microbial state tested and the biological endpoint measured [37,41].

### 4.3. Drug-Resistant Pathogens as Biological Stress Tests

Drug-resistant pathogens provide demanding test conditions for antimicrobial biomaterials because resistance phenotypes can coexist with biofilm formation, persistence, and physiological adaptation. The 2024 WHO Bacterial Priority Pathogens List classifies CRAB as a critical priority pathogen and CRPA and MRSA as high-priority pathogens [1,18]. In this review, these three organisms are selected as complementary biological stress tests rather than as representative organisms for all drug-resistant or biomaterial-associated infections.

Importantly, resistance status should not be treated as a proxy for biofilm formation or other strain-level biological traits. These properties can vary among clinical isolates and should be measured rather than inferred from the resistance designation alone [42,43]. Their selection provides contrasting Gram-negative and Gram-positive challenges involving resistance, surface-associated growth, persistence, and adaptation.

#### 4.3.1. Carbapenem-Resistant *Acinetobacter baumannii*

CRAB represents a clinically important resistant Gram-negative challenge and is classified by the WHO as a critical priority pathogen [1]. Its inclusion allows antimicrobial biomaterials to be considered across different Gram classifications and clinically relevant patterns of biofilm formation, persistence, and adaptation. *A. baumannii* can survive in healthcare environments and form biofilms on abiotic surfaces, characteristics that contribute to its persistence and relevance to healthcare-associated infection [44]. Clinical studies also demonstrate substantial variation among *A. baumannii* isolates in both AMR and biofilm formation, indicating that these phenotypes should be measured rather than assumed from species or resistance status alone [42,45]. CRAB provides a useful stress test for assessing whether a biomaterial retains antimicrobial activity against resistant, surface-associated bacterial populations while accounting for biological variation among clinical isolates. Carbapenem resistance should not, however, be treated as synonymous with multidrug resistance or strong biofilm formation in every isolate [45,46].

#### 4.3.2. Methicillin-Resistant *Staphylococcus aureus*

MRSA provides a complementary Gram-positive challenge with direct relevance to biomaterial-associated infection. *S. aureus* is a major cause of implant-associated infection, and biofilm formation promotes bacterial persistence and protection from antimicrobial treatment and host defenses [2]. For antimicrobial biomaterials, MRSA therefore tests more than direct antibacterial activity. Materials intended for implant applications should limit bacterial colonization while preserving host–cell compatibility and tissue integration [14].

#### 4.3.3. Carbapenem-Resistant *Pseudomonas aeruginosa*

CRPA provides a complementary Gram-negative challenge in which biofilm-associated tolerance and physiological adaptation are important considerations. The WHO classifies CRPA as a high-priority pathogen, and *P. aeruginosa* biofilms can contain metabolically distinct populations with different responses to antimicrobial exposure [5,40]. Experimental studies further show that *P. aeruginosa* can adapt to host environments and that biofilm formation can alter antibiotic tolerance during infection [47]. CRPA therefore provides a useful context for asking whether antimicrobial activity remains effective against a resistant population whose physiology can change during prolonged infection.

CRAB, MRSA, and CRPA provide complementary biological stress conditions for evaluating antimicrobial biomaterials. CRAB provides a context for examining resistant Gram-negative, surface-associated infection; MRSA provides a clinically relevant Gram-positive context for implant-associated colonization and biofilm formation; and CRPA provides a context for examining biofilm-associated tolerance and adaptation. Their selection therefore creates a focused testing framework without requiring separate pathogen reviews. Table 1 summarizes the complementary clinical and biological challenges represented by CRAB, MRSA, and CRPA and defines the corresponding performance criteria for antimicrobial biomaterials.

#### 4.3.4. *L. monocytogenes* as a Food-Industry Biofilm Challenge

In food-processing environments, *L. monocytogenes* can form persistent biofilms on food-contact surfaces and processing equipment, creating a continuing risk of contamination and cross-contamination. Its persistence has been associated with surface attachment, biofilm formation, environmental adaptation, and survival under cleaning and disinfection conditions. These characteristics make *L. monocytogenes* an important additional example of a biofilm-forming pathogen when evaluating antimicrobial surfaces for food-industry applications [49].

Although the present review focuses primarily on medical device- and implant-associated infections, inclusion of *L. monocytogenes* broadens the biological context of Part 4 by recognizing the importance of biofilm control in food-processing environments. It should therefore be considered a distinct food-industry challenge rather than another representative of the CRAB, MRSA, and CRPA stress-test group.

### 4.4. From Reference Strains to Clinically Relevant Infection Models

Reference strains provide standardized organisms that improve reproducibility and facilitate comparisons among experiments. They are useful for initial screening of antimicrobial activity under controlled conditions, particularly when comparing material formulations or surface modifications. Their limitations become important when findings are extended toward clinical translation. Clinical isolates can differ in biofilm formation and other phenotypic characteristics from commonly used laboratory strains, as demonstrated for *P. aeruginosa* and *S. aureus* [43,50].

Testing resistant clinical isolates adds another level of biological relevance because resistance and biofilm phenotypes can vary among isolates. In *A. baumannii*, clinical studies have demonstrated variation in both AMR and biofilm formation, supporting direct characterization of the isolate rather than inference from its resistance designation alone [42,45]. The next step is to evaluate established biofilms rather than relying only on planktonic growth or early attachment. Biofilm models can measure biomass, viable cell reduction, minimum biofilm inhibitory concentration, and minimum biofilm eradication concentration, but differences among experimental systems can substantially influence measured antimicrobial efficacy [36,37,41].

Greater biological fidelity can then be introduced by incorporating features of the intended infection environment. Depending on the application, these may include physiological fluids, host proteins, tissue components, three-dimensional matrices, immune cells, or other relevant environmental conditions. Recent work on chronic wound models emphasizes that increasing model complexity can improve representation of host and microbial interactions, while no single in vitro system can reproduce the full complexity of chronic infection [51].

**Figure 2:**
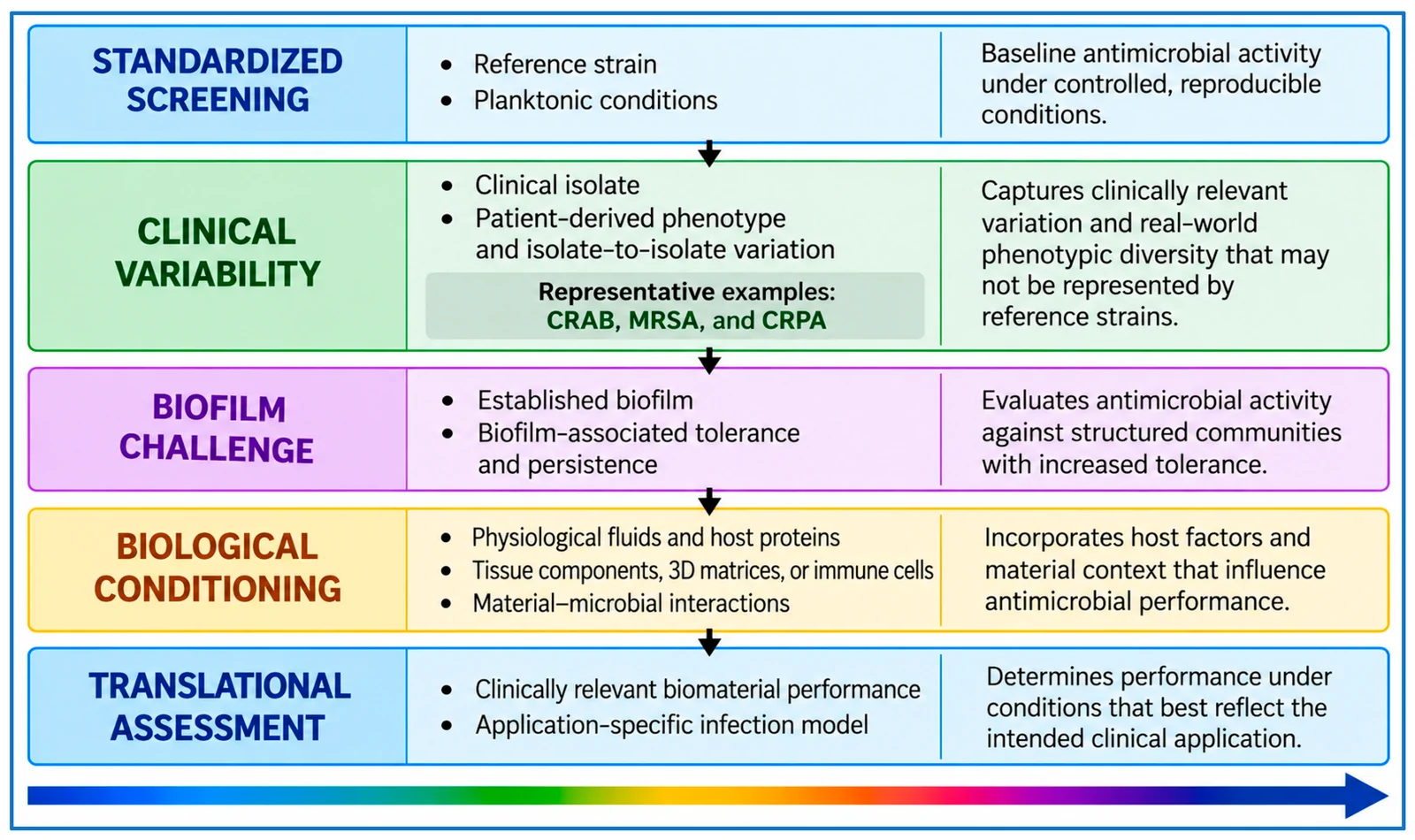
Progression from standardized screening to translational evaluation of antimicrobial biomaterials. The framework moves from reference strains and controlled planktonic conditions through clinical isolates, established biofilms, biological conditioning, and application-specific infection models, with CRAB, MRSA, and CRPA representing selected resistant pathogen stress-test examples.

This progression should not be interpreted as a requirement to use the most complex model at every stage. Model complexity should be matched to the scientific question and the level of biological relevance required. Reference strains are appropriate when standardized and reproducible conditions are needed, whereas clinical isolates may be appropriate when isolate-level variation or clinical relevance is the focus [36,37,38]. Resistant clinical isolates should also be phenotypically characterized rather than classified solely according to their resistance designation. Accordingly, the choice of strain and model should be guided by the specific scientific question rather than by an assumption that clinical isolates are inherently superior to reference strains. Figure 2 illustrates this progression from standardized screening to increasingly biologically relevant evaluation of antimicrobial biomaterials.

The central testing principle is therefore that strong activity against a laboratory strain under planktonic conditions does not establish robust performance against a clinically relevant resistant biofilm under physiologically relevant conditions. Meaningful translational assessment requires evaluation across relevant microbial states and biological environments rather than reliance on a single experimental model [38].

## 5. Preventing Colonization: Engineering the Interface Before Infection Is Established

### 5.1. Antifouling and Antiadhesive Strategies

A zwitterionic poly(sulfobetaine methacrylate) coating grafted onto Ti6Al4V implant surfaces reduced *S. aureus* adhesion. In a mouse implant infection model, the coating combined with a single vancomycin injection also reduced bacterial colonization and periprosthetic infection. This finding illustrates how a hydrated, low-fouling surface can limit bacterial attachment before stable colonization develops [52]. Antifouling interfaces generally reduce nonspecific interactions by creating highly hydrated or sterically restrictive surface layers that limit close contact between microorganisms and the material [53,54]. Polymeric antifouling layers can retain interfacial water and restrict molecular approach through steric effects. Poly(ethylene glycol) (PEG) is a widely studied example, although oxidative degradation, fouling, and other stability limitations can compromise long-term performance [54,55].

Zwitterionic polymers offer another antifouling strategy. Their combination of closely spaced positive and negative charges promotes strong hydration while maintaining overall charge neutrality, which can reduce nonspecific protein and microbial adsorption [56]. Antifouling performance is also influenced by polymer architecture, including chain organization, surface coverage, and coating structure. Thus, polymer chemistry alone does not determine the behavior of the final interface [54,57]. A reduction in initial bacterial adhesion should not, by itself, be described as prevention of biofilm formation unless subsequent stages of biofilm development are also evaluated [53,58].

### 5.2. Engineering Surface Properties to Reduce Initial Attachment

Different nanoscale architectures on titanium surfaces produced different attachment patterns for *S. aureus* and *P. aeruginosa*. Small changes in surface architecture altered *P. aeruginosa* colonization even when the average surface roughness was very low. This finding shows that bacterial attachment cannot be predicted from average roughness alone [59]. 

Surface charge, wettability, surface free energy, roughness, and topography can influence bacterial attachment, but their effects depend on the microorganism, material, surrounding medium, and experimental conditions. No single physicochemical parameter provides a universal predictor of bacterial adhesion [60,61]. Surface charge can modify electrostatic interactions between bacterial cells and materials, whereas wettability is influenced by both surface chemistry and topography. Contact angle measurements should therefore be interpreted as one component of surface characterization rather than as a stand-alone predictor of microbial adhesion [62].

Roughness and topography can alter bacterial contact, adhesion forces, and the local environment at the material surface. Importantly, average roughness does not fully capture three-dimensional surface architecture, and patterned or nanoscale features can produce different adhesion outcomes depending on their geometry and dimensions [24,58]. These findings favor integrated surface design rather than optimization of a single parameter. This consideration is especially relevant for implants because changes introduced to limit bacterial attachment can also alter interactions with mammalian cells [24,63].

### 5.3. Blocking the Transition from Attachment to Colonization

A recent study provides a direct example of this distinction using a clinical MRSA isolate. PEGylated poly-D,L-lactide coatings on titanium reduced biofilm formation and altered biofilm structure, indicating that the coating affected both early bacterial attachment and subsequent development of the biofilm community [64]. Reducing initial attachment does not necessarily prevent subsequent biofilm development. A preventive interface must limit the progression from reversible contact to stable adhesion and early surface colonization [53,58]. Once stable attachment occurs, bacteria can develop microcolonies and produce extracellular polymeric substances that strengthen the developing community. Preventive strategies are most useful when they interrupt this transition before stable colonization is established [53].

Antiadhesive activity should nevertheless not be interpreted as complete prevention of biofilm formation. Adhesion remains context dependent, and a surface that limits attachment under one experimental condition may perform differently with another organism or biological environment [24,60]. Therefore, antiadhesive studies should, where relevant, include longer incubation periods or subsequent biofilm-development assays in addition to short-term measurements of bacterial attachment [53,58].

### 5.4. Balancing Microbial Repellency with Host Integration

DNA-chitosan multilayer coatings on titanium reduced *S. aureus* and *S. epidermidis* adhesion and biofilm growth while supporting osteoblast-like cell attachment and spreading. The findings show that an implant surface can be designed to reduce bacterial fouling without necessarily preventing the host–cell interactions required for tissue integration [65].

A major design challenge for implantable antimicrobial surfaces is achieving microbial exclusion without suppressing the cellular interactions required for tissue integration. Strongly nonadhesive surfaces can reduce bacterial attachment but may also restrict mammalian cell adhesion and spreading [63,65].

Thus, the relevant design objective is selective interfacial control rather than maximal suppression of surface interactions. For implantable materials, antimicrobial prevention should be considered together with the cellular responses required for tissue integration [63,65].

### 5.5. Preserving Preventive Function After Biological Exposure

Serum albumin preadsorption on polydimethylsiloxane changed bacterial attachment in a species-dependent manner. It reduced initial attachment of *S. aureus* and *S. epidermidis*, but prolonged exposure promoted *P. aeruginosa* attachment and biofilm formation. These findings show why preventive performance should be evaluated after biological conditioning rather than only on pristine surfaces [13]. The preventive function of a surface should be evaluated after relevant biological conditioning because protein adsorption and other interfacial changes can alter the properties encountered by microorganisms [13,56]. 

Preventive performance should therefore be assessed after relevant biological conditioning rather than inferred exclusively from measurements obtained with pristine materials. This is particularly important for polymeric coatings whose function depends on surface hydration, molecular organization, or accessibility [54,57]. Long-term stability presents an additional constraint. Antifouling coatings must retain their surface organization and functional properties during the period of use, while degradation, mechanical damage, swelling, or loss of coating integrity can reduce performance.

Accordingly, an antiadhesive surface should be evaluated not only by its initial reduction in bacterial attachment but also by its ability to retain that function after relevant biological exposure and material aging. This shifts the performance criterion from initial antiadhesion to durable prevention of colonization [55,66]. Durable prevention therefore requires an interface that retains anti-colonization function after biological conditioning while preserving the host interactions required for tissue integration [63,65].

## 6. Active Antimicrobial and Antibiofilm Strategies: Beyond Conventional Bacterial Killing

Once microbial colonization is established, antimicrobial biomaterials require active strategies that address the microbial population already present at the interface. Current approaches include direct membrane targeting, localized delivery of antimicrobial agents, externally activated or catalytic mechanisms, and strategies that disrupt biofilm structure or alter bacterial behavior. Their effectiveness depends on the biological state being targeted and on whether the intended outcome is bacterial killing, biofilm disruption, or sustained control of infection [67,68].

### 6.1. Membrane Directed and Molecular Antimicrobial Mechanisms

Tet213, an antimicrobial peptide (AMP), was incorporated into a titanium coating and tested against *S. aureus* isolates obtained from implant-associated infections. The peptide reduced bacterial growth and biofilm formation, but the response varied among clinical isolates. The findings illustrate how a membrane-active peptide can provide local antimicrobial activity while also highlighting the importance of isolate-level evaluation [69]. 

AMPs and cationic polymers can interact preferentially with bacterial cell envelopes through electrostatic and hydrophobic interactions. Many AMPs are cationic and amphipathic, enabling association with bacterial membranes and, for some peptides, membrane permeabilization or disruption; however, their mechanisms vary substantially with peptide sequence, structure, and bacterial target [70,71]. AMP-based biomaterials have been developed in several formats, including coatings, hydrogels, electrospun materials, self-assembled systems, and localized delivery platforms. Incorporating AMPs into biomaterials can improve local retention, stability, or controlled exposure compared with administration of the free peptide, although these advantages vary with the material design and intended application [70,71].

Cationic polymers use related electrostatic interactions and can be engineered to produce contact-mediated antimicrobial activity. Their antibacterial effects can involve interaction with bacterial envelopes, membrane disruption, and leakage of intracellular components. Charge switchable polymers provide a related strategy in which environmental cues can alter polymer charge and, consequently, its interaction with bacterial cells and biofilms [68]. These mechanisms should not be considered universal, because the antimicrobial effects of peptides and polymers can vary with molecular structure, charge, hydrophobicity, and the bacterial target [68,70,71].

The main limitation is that strong membrane activity does not necessarily translate into selective antimicrobial activity or adequate host compatibility. Cationic polymers can cause membrane perturbation and cytoplasmic leakage, but their antibacterial effects must be considered alongside interactions with mammalian cells. Potential cytotoxicity and other effects on mammalian cells should therefore be evaluated alongside antibacterial activity, particularly for strongly cationic or membrane-active materials [68,70]. For AMPs, peptide stability, proteolytic degradation, surface accessibility, and interactions with biological components can also influence activity and therapeutic performance [68,70]. For biomaterial design, the relevant objective is therefore not maximal membrane disruption, but effective bacterial targeting with adequate host compatibility and sustained activity at the intended interface.

### 6.2. Localized Delivery and Reactive or Physically Activated Antimicrobial Mechanisms

A collagen-based hydrogel loaded with rifampin was used to treat MRSA osteomyelitis in a mouse fracture model. The high-dose hydrogel reduced MRSA recovered from both tissue and implants and improved fracture healing, although combined local and systemic treatment was more effective than local delivery alone. This finding illustrates how a biomaterial can provide local antibiotic exposure while showing that release from the material does not necessarily replace systemic treatment [72].

Localized delivery systems can concentrate antimicrobial agents at or near an infected or infection-prone site and can, depending on the application, reduce reliance on systemic exposure. Antibiotic-loaded biomaterials have been investigated using polymeric carriers, hydrogels, bone substitutes, and other local delivery platforms. Depending on the material design, release can occur through diffusion, material degradation, or externally or biologically triggered mechanisms [73,74].

The effectiveness of local delivery depends on maintaining therapeutically relevant antimicrobial exposure at the target site for the required period, with release kinetics determining the resulting exposure profile [73,74]. Rapid release can produce high initial concentrations but may lead to early depletion, whereas prolonged release does not necessarily ensure adequate antimicrobial exposure throughout the treatment period. Material degradation, tissue distribution, and local biological conditions can further modify the resulting exposure profile [73,74].

Metal ions, including silver and other antimicrobial metals, have been incorporated into biomaterials to deliver localized antibacterial activity. Their effects can involve multiple cellular targets, while antimicrobial performance and host compatibility depend on factors such as ion concentration, release behavior, material composition, and exposure conditions [67].

Reactive and physical approaches provide additional mechanisms that do not rely exclusively on conventional antibiotic targets. A titanium surface coated with MoS_2_ and the photosensitizer IR780 combined photothermal and photodynamic activity under near-infrared irradiation and reduced an established *S. aureus* biofilm in vivo. This finding illustrates how externally activated biomaterials can act against an established biofilm through more than one physical mechanism [75].

Photodynamic antimicrobial therapy uses a photosensitizer activated by light to generate ROS, whereas photothermal approaches convert absorbed light into localized heat. Both approaches have been investigated for antibacterial and biofilm-related applications, but their performance depends on factors including light delivery, material architecture, oxygen availability for photodynamic action, and treatment conditions [72,73].

Catalytic systems provide another route to antimicrobial activity at the material interface. Nanozymes can exhibit enzyme-like catalytic activity and have been investigated for antibacterial applications, including systems that generate reactive species or combine catalytic activity with other antimicrobial mechanisms. Their performance is influenced by the catalytic material and the chemical conditions of the surrounding environment [76,77].

Translation of these approaches requires evaluation under conditions that reflect the intended application rather than relying solely on activity demonstrated in simplified laboratory systems. Relevant considerations include stimulus availability for externally activated systems, oxygen availability for photodynamic processes, substrate availability for catalytic systems, release behavior for delivery systems, and potential effects on surrounding tissue [67,76]. 

For photodynamic systems, studies should report the photosensitizer concentration, wavelength, irradiance, radiant exposure, and irradiation time, together with relevant experimental controls and oxygen conditions. These parameters are important for reproducibility and comparison between studies [78,79]. For photothermal systems, studies should report the wavelength, power density, irradiation time, and treatment temperature, together with assessment of host–cell or tissue safety [80,81].

### 6.3. Biofilm Directed and Non-Antibiotic Biological Strategies

A DNase-loaded AuAgCu hydrogel was developed to disrupt MRSA biofilms by degrading extracellular DNA within the biofilm matrix. The hydrogel was designed to improve penetration of reactive species and heat into the biofilm, and it reduced bacterial burden in an MRSA-infected wound model while showing good biocompatibility. This finding illustrates how targeting the biofilm matrix can improve access to bacteria that are otherwise protected within the community [82].

Established biofilms provide targets beyond bacterial viability. Selected components of the EPS matrix can be targeted to alter biofilm structure and accessibility to subsequent antimicrobial treatment [83]. Extracellular DNA (eDNA) is an important matrix component of the biofilm matrix and can contribute to its structural organization and stability. Matrix-degrading enzymes and other approaches targeting extracellular components have been investigated as adjunctive antibiofilm strategies. Their effects remain dependent on the composition of the matrix and the bacterial species involved [68,83].

For example, a multifunctional surface containing a quorum-sensing (QS) inhibitor and nitric oxide reduced colonization by *S. aureus* and *P. aeruginosa* without relying solely on bacterial killing. This illustrates how a biomaterial can interfere with bacterial communication while providing another antimicrobial function at the same interface [84]. QS interference represents a complementary approach to direct antimicrobial killing. It targets bacterial communication systems that regulate collective behaviors, including biofilm development and virulence, rather than directly targeting bacterial viability. Its therapeutic value depends on whether disruption of the targeted signaling pathway produces meaningful reduction in biofilm formation, virulence, or infection persistence [85].

For example, bacteriophages have been tested against multidrug-resistant *P. aeruginosa* biofilms on catheter and endotracheal tube surfaces. In one study, phage-coated segments reduced biofilm formation and bacterial counts compared with untreated surfaces. This example illustrates the potential of phage-based biomaterials to target resistant bacteria directly at device surfaces [86]. Bacteriophages provide another non-antibiotic strategy through infection and lysis of susceptible bacterial hosts. Phage-derived enzymes, including endolysins and matrix-degrading enzymes, can provide complementary mechanisms by damaging bacterial cell envelopes or disrupting biofilm-associated structures. Engineered phages and phage-derived enzymes have therefore been investigated against biofilms formed by antibiotic resistant bacteria, with experimental studies reporting enhanced biofilm disruption and bacterial killing in selected systems [87].

The specificity of bacteriophages is both a potential advantage and a practical limitation. Their activity depends on the susceptibility and receptor availability of the target bacterium, while narrow host range, phage resistance, delivery, stability, and access to bacteria within established biofilms remain important considerations. These constraints support considering phage-based approaches as complementary or combination strategies in many applications rather than as universally applicable replacements for conventional antimicrobials [67,87].

These approaches broaden the range of targets available for established biofilms beyond bacterial viability alone, including matrix structure, bacterial signaling, and susceptible bacterial populations [83,85,87]. Biofilm assays should be interpreted according to the biological variable measured. Crystal violet primarily measures attached biomass, whereas metabolic assays measure metabolic activity. Colony-forming units (CFU) enumeration measures culturable cells recovered from the biofilm. Microscopy provides spatial information, and regrowth assays assess surviving populations [37].

The outcomes produced by active antimicrobial biomaterials should not be treated as interchangeable. Killing planktonic bacteria, reducing an established biofilm, and preventing recurrence are distinct biological endpoints and require different evidence. Reduction in biofilm biomass or metabolic activity alone does not demonstrate eradication of an established biofilm [37].

Therefore, the central design challenge is to match the antimicrobial mechanism and performance criteria to the biological state of the infection. Biomaterials should be evaluated not only for antimicrobial potency but also for whether their activity is accessible, sustained, host-compatible, and durable at the intended site.

## 7. Adaptive Biomaterials: Detecting Infection and Responding at the Right Time and Place

Once antimicrobial function has been incorporated into a biomaterial, an important design question is when and where that function should operate. Constitutively active materials can provide continuous protection, but sustained activity may also increase unnecessary exposure of surrounding tissue or consume active components when additional treatment is not required. Adaptive biomaterials seek to modulate antimicrobial activity in response to endogenous features of the local environment or externally applied stimuli. Their value therefore depends not simply on responsiveness, but on the selectivity, controllability, and fit of the resulting response to the biological context [88,89,90]. Responsive materials are not inherently superior to continuously active materials. The appropriate strategy depends on the clinical application and the required duration and context of antimicrobial protection [88,89,90].

### 7.1. Why and When Should Antimicrobial Activity Be Activated?

A multilayer coating containing gentamicin was designed to remain relatively stable under physiological conditions but release the antibiotic when exposed to hyaluronidase or a bacterial infection environment. The coating showed greater gentamicin release in the presence of *Escherichia coli* (*E. coli*) than in phosphate-buffered saline and provided prolonged antibiofilm activity. This finding illustrates the rationale for keeping antimicrobial activity relatively low under baseline conditions while increasing it when an infection-associated signal is present [91]. 

Continuous antimicrobial activity is not inherently undesirable. For preventive applications, persistent surface activity may be appropriate because protection is required before an infection-associated signal is present. Thus, continuous activity may be appropriate when sustained protection is the primary requirement. The rationale for adaptive activation arises when additional antimicrobial activity carries potential costs, including unnecessary host exposure and depletion of active agents. Responsive systems therefore seek to distinguish baseline material function from additional antimicrobial activity activated under defined conditions [88,90].

This distinction is particularly relevant to localized delivery. A continuously releasing material provides sustained exposure but does not inherently adjust release according to changes in the local biological state. Infection-responsive systems instead couple antimicrobial release or activation to a detectable environmental change, allowing activity to be regulated according to the stimulus and material response kinetics [88,89]. Hence, the design objective is not maximal responsiveness. An adaptive material should respond to a biologically meaningful signal, generate sufficient antimicrobial activity, and minimize inappropriate activation. Stimulus-responsiveness alone is consequently insufficient to establish biological usefulness. 

### 7.2. Infection-Associated Signals and Activation Mechanisms

A pH-responsive coating was developed on titanium by incorporating the AMP HHC36 into a chitosan and polydopamine-based surface. Under physiological conditions, the coating limited peptide exposure, whereas the acidic environment associated with bacterial infection promoted peptide release and antimicrobial activity. The coating also supported cell compatibility and osteogenic responses, illustrating how an infection-associated signal can be linked to both antimicrobial activation and implant function [92].

Endogenous signals can provide triggers for coupling antimicrobial activity to changes in the local infection environment. Frequently investigated cues include pH, bacterial enzymes, ROS, and metabolic or redox changes. These signals can be incorporated into material designs through responsive chemical bonds, ionizable groups, degradation mechanisms, or other architectures that alter antimicrobial release or activity when the surrounding environment changes [88,89].

Acidic microenvironments associated with bacterial metabolism have been exploited in pH-responsive systems to regulate antimicrobial release or surface properties. Enzyme-responsive materials provide another approach by using enzymes associated with bacterial activity as triggers for chemical cleavage or changes in material structure. However, neither pH nor enzyme activity is universally specific to infection. Local pH can vary with tissue metabolism and inflammation, while enzymes may originate from host as well as microbial processes [88,90].

ROS provide another potential trigger because oxidative conditions occur in inflammatory and infectious microenvironments. ROS-responsive materials can undergo chemical or structural changes under oxidative conditions, enabling stimulus-dependent drug release or other changes in material function. Their selectivity is nevertheless limited by the physiological and inflammatory roles of ROS, making the magnitude, duration, and spatial distribution of the signal important considerations [93].

Metabolic and redox changes have likewise been investigated as endogenous cues for responsive antimicrobial systems. Their usefulness is determined in part by whether the selected signal can distinguish microbial activity from changes generated by host metabolism or tissue injury [88,89].

Inflammatory signals present a particularly important limitation. Infection activates innate immune pathways, but implantation and tissue injury can also produce sterile inflammation through damage-associated signals. An inflammatory environment cannot be assumed to indicate infection. Responsive systems that use inflammatory conditions as triggers must be tested for activation specificity using relevant noninfectious controls, including sterile inflammation or tissue damage where appropriate. The usefulness of an endogenous trigger ultimately depends on its specificity, threshold, and relationship to the local biological state [94].

### 7.3. Controlling Where and When the Response Occurs

Ultrasound-activatable nanoparticles containing meropenem were designed to target *P. aeruginosa* biofilms. Ultrasound triggered phase transition and cavitation, which disrupted the biofilm matrix and promoted local antibiotic release. The system showed antibiofilm activity in vitro and anti-infective effects in vivo. This approach illustrates how an external stimulus can control both the timing of activation and access to bacteria within an established biofilm [95].

Adaptive antimicrobial systems provide two complementary forms of control: spatial control, which determines where antimicrobial activity is generated, and temporal control, which determines when and for how long it occurs. Combining these dimensions can concentrate activity at the target site while limiting exposure outside the intended treatment period [89,90].

Spatial control is particularly relevant when antimicrobial activity may affect surrounding tissue. Local activation can reduce exposure outside the target region, but its effectiveness depends on stimulus distribution and whether the activated material or released agent can reach bacteria throughout the infected site. Activation at the material surface does not necessarily ensure that the active compound reaches all regions of an established biofilm. Antimicrobial penetration within biofilms can be affected by diffusion, sorption, chemical reaction, and the physicochemical properties of the antimicrobial agent. Biofilms also contain spatially heterogeneous bacterial populations and local microenvironments, which can contribute to differences in antimicrobial exposure and response [37,96].

Temporal control introduces a related requirement. An adaptive system should ideally activate when the relevant trigger reaches the required threshold, maintain sufficient activity for the intended treatment period, and decrease activity when the stimulus falls below that threshold. In practice, material kinetics, diffusion, degradation, and persistence of the triggering signal may not follow the biological course of infection. Responsive behavior should therefore be measured experimentally rather than inferred from the presence of a stimulus-responsive component [88,89].

Externally controlled systems provide a different form of regulation. Light, ultrasound, magnetic fields, and other physical stimuli can permit activation at a selected time without requiring the material to detect an endogenous infection signal. They can offer substantial control over treatment, but their clinical usefulness depends on the ability to deliver the stimulus safely and effectively to the target site. Light-based approaches can be limited by tissue penetration, whereas ultrasound and magnetic systems require appropriate equipment and adequate energy transfer at the target location [89,97].

Photodynamic and photothermal systems illustrate these constraints. Photodynamic therapy requires light delivery and molecular oxygen to generate ROS, whereas photothermal therapy converts absorbed light into heat. Both approaches can provide externally controlled antimicrobial activity, but treatment depth, illumination geometry, energy exposure, and potential tissue effects remain important considerations. Recent work on near-infrared II systems has sought to improve tissue penetration, although clinical translation remains an active area of investigation [98].

**Figure 3:**
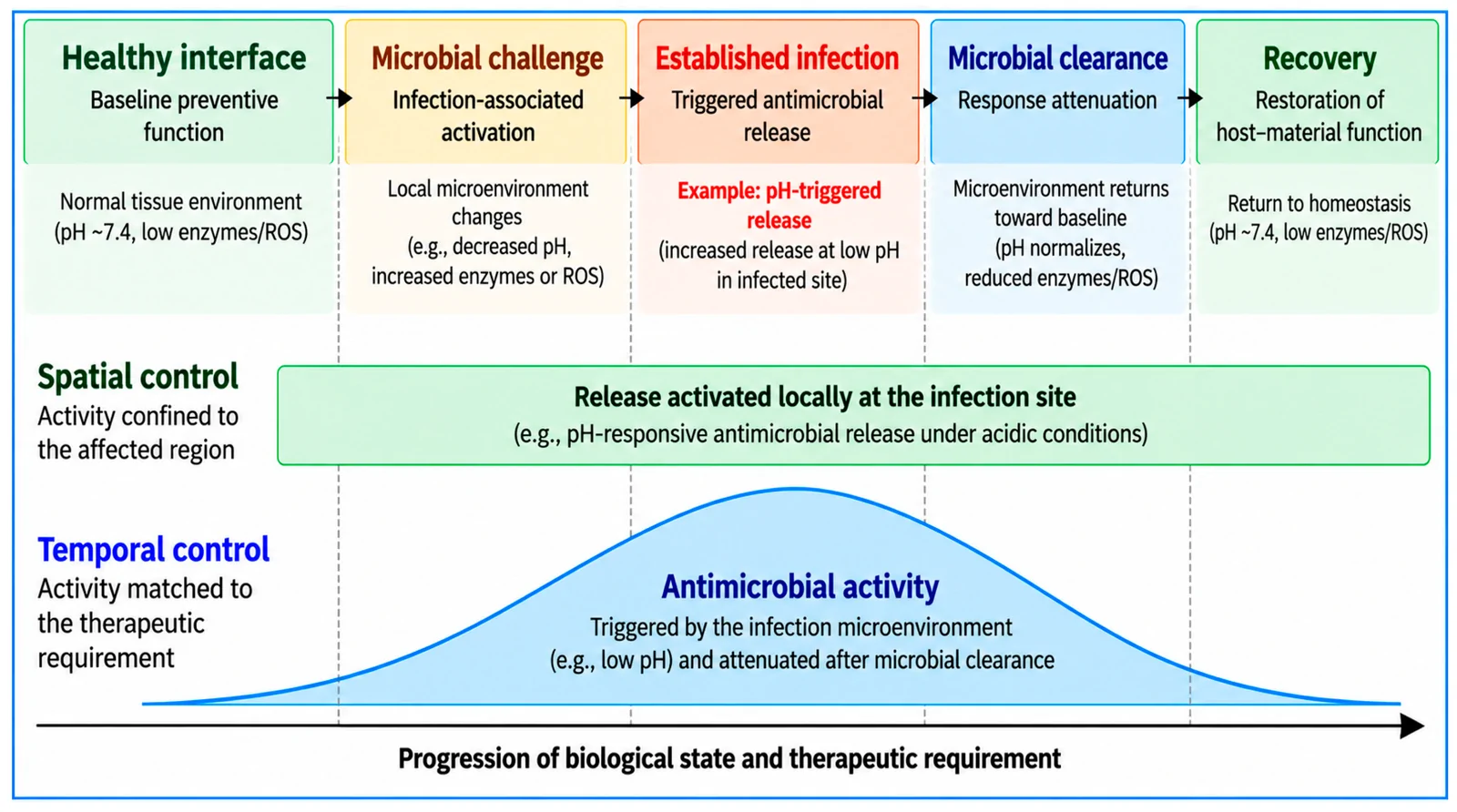
Temporal and spatial programming of infection-responsive biomaterials. The schematic illustrates localized and temporally controlled antimicrobial activity during microbial challenge, established infection, microbial clearance, and recovery. A pH-triggered release example illustrates how antimicrobial activity can be activated under infection-associated conditions and attenuated after microbial clearance. The trajectory is conceptual and does not represent a universal or experimentally established response profile.

Endogenous and external activation should therefore be compared according to clinical function rather than technological novelty. Endogenous systems provide autonomous responses to local biological conditions, whereas external systems allow deliberate control over treatment timing and, in some systems, location. Their suitability depends on infection accessibility, trigger reliability, required treatment intensity, and safety. Figure 3 illustrates how spatial and temporal control can be integrated across changing biological states, from baseline protection through microbial challenge and established infection to clearance and recovery.

### 7.4. From Stimulus-Responsive to Biologically Appropriate Interfaces

A recent titanium implant coating illustrates this distinction. The coating showed pH-responsive antimicrobial behavior while maintaining biocompatibility and osteogenic potential. This example shows why an adaptive interface should be evaluated not only for stimulus responsiveness, but also for whether the response is appropriate for the intended biological environment [99].

Stimulus-responsiveness alone does not establish that a biomaterial is biologically adaptive. In this review, “detect” refers to the material recognizing an infection-associated signal that triggers or modulates its antimicrobial activity; it does not imply diagnostic detection or confirmation of infection. A material may respond reliably to pH, enzymes, ROS, light, or another stimulus while activating at an unsuitable threshold, location, or duration. A measurable response to a stimulus does not establish that the stimulus is specific to infection or that the resulting antimicrobial activity is therapeutically sufficient. The relevant criterion is whether the response is appropriately matched to the biological state that the material is intended to manage [88,90].

This consideration is especially relevant when a selected stimulus can also arise during noninfectious tissue responses. Implantation and tissue injury can generate inflammatory and oxidative conditions that overlap with features of infection. A responsive material that cannot distinguish these states may therefore activate antimicrobial functions unnecessarily. Specificity should consequently be evaluated against relevant noninfectious controls rather than against an untreated healthy condition alone [93,94].

A biologically appropriate adaptive interface should provide a controlled progression from baseline protection to additional antimicrobial activity when an infection-associated signal is sensed, followed by attenuation when the relevant stimulus decreases. Conceptually, this corresponds to a healthy interface with baseline preventive function, localized activation during microbial challenge, intensified antimicrobial or antibiofilm activity when established infection requires intervention, and reduced additional activity as the infectious stimulus resolves. The precise sequence will depend on the material and application and should therefore be regarded as a design objective rather than a universal biological program.

The most informative evaluation criteria are trigger specificity, activation threshold, spatial precision, response kinetics, therapeutic sufficiency, host compatibility, and durability. These criteria shift the assessment of adaptive biomaterials from simple stimulus-responsiveness toward controlled antimicrobial function appropriate to the biological context.

## 8. Host Response and Tissue Recovery: From Infection Control to Functional Healing

Effective antimicrobial activity does not by itself establish successful biomaterial performance. Infection develops within a host environment in which immune responses, inflammation, tissue repair, and material-tissue interactions jointly influence the outcome. Biomaterials can modulate this environment through their physicochemical properties, degradation behavior, and presentation or delivery of biological signals. The objective is to support effective host defense while limiting excessive or prolonged inflammation that could impair tissue repair [100,101,102].

### 8.1. Coordinating Antimicrobial Activity with Host Defense

A thermosensitive hydrogel containing the AMP GL13K was applied to titanium surfaces and tested under conditions relevant to infected implants. The system provided prolonged AMP release and showed antibacterial activity while also reducing inflammatory responses and increasing markers associated with a reparative macrophage phenotype. These findings illustrate how an antimicrobial implant surface can influence both bacterial control and the local immune response [103].

Innate immunity is an essential component of infection control. Neutrophils contribute to early antimicrobial defense, while macrophages participate in phagocytosis, inflammatory regulation, debris clearance, and tissue remodeling. Their functions are dynamic and shaped by the local tissue environment and stage of the response rather than following a fixed sequence [100].

For biomaterials, immune compatibility extends beyond the absence of acute cytotoxicity. Material properties can influence macrophage recruitment and function, with consequences for inflammation, vascularization, and tissue remodeling [100,104]. Macrophage behavior should not, however, be reduced to a simple M1/M2 transition. These categories remain useful experimental descriptors, but they do not capture the heterogeneity and functional plasticity of macrophages in vivo [100,105].

Microbial control must therefore remain compatible with effective host defense. Inflammation is necessary for antimicrobial defense and tissue repair, but persistent or dysregulated inflammation can delay healing and contribute to fibrosis and impaired tissue function [101,102]. The relevant design objective is not generalized immune suppression, but appropriate regulation of the host response.

### 8.2. Linking Infection Resolution to Tissue Repair

A degradable three-layer coating on titanium combined vancomycin delivery with local release of interleukin-12 (IL-12). The coating reduced MRSA growth and was tested in a rat tibial implant infection model, where local IL-12 delivery was used to modify the immune response during infection. The coating also showed cell-binding properties that supported implant integration. This approach illustrates how antimicrobial treatment can be combined with local immune regulation rather than treating infection and tissue repair as separate problems [106].

Microbial clearance does not by itself restore damaged tissue. Tissue repair depends on coordinated resolution of inflammation, extracellular-matrix remodeling, vascular responses, and tissue-specific regenerative processes [100]. Biomaterial-based immunomodulation has consequently been investigated as a means of influencing the local environment during tissue repair, including in vascular and bone regeneration [104,107,108].

The relationship between immune regulation and regeneration is well illustrated in vascular and bone repair. Biomaterial properties can shape macrophage behavior associated with vascular formation and tissue remodeling [104]. In bone regeneration, immune cells and local immune signals interact with bone-forming cells and can influence repair, while biomaterial-based immunomodulation has been investigated to improve the osteogenic environment [107,108]. In wound applications, responsive antimicrobial biomaterials have also been investigated to combine microbial control with tissue repair [109].

Antimicrobial efficacy and regenerative performance are distinct outcomes. Reduced bacterial burden does not by itself demonstrate improved tissue repair, which requires evidence of appropriate tissue-specific healing and restoration. The design objective is therefore to achieve microbial control without creating material- or host-mediated responses that compromise subsequent repair [110,111].

### 8.3. From Infection Control to Functional Recovery

A ROS-responsive hyaluronic acid/polyvinyl alcohol hydrogel was designed to deliver moxifloxacin and curcumin sequentially in MRSA-infected wounds. In a mouse wound model, treatment reduced bacterial burden and inflammation and increased collagen deposition and vascular regeneration, together with faster wound closure. These findings show why assessment of an antimicrobial biomaterial should extend beyond bacterial reduction to include tissue repair and restoration of wound structure [112].

The final outcome of an antimicrobial biomaterial should extend beyond reduction in microbial burden. Successful treatment should permit progression from infection control through resolution of inflammation and tissue restoration to recovery of tissue-specific function, where relevant [100,101].

Wound treatments may require restoration of tissue continuity and barrier function, whereas implant applications may require stable host-material integration. Bone and other tissue-engineering applications may additionally require restoration of structural and biological function. Where regeneration depends on adequate perfusion, vascularization is an additional relevant outcome [104]. 

Importantly, successful microbial control does not guarantee a favorable tissue outcome. Persistent inflammation, excessive foreign-body responses, fibrosis, or inadequate tissue remodeling can compromise recovery despite effective antimicrobial activity [100,101]. Therefore, cytotoxicity testing alone is insufficient to establish host compatibility. Biological evaluation should be matched to the intended application and duration of contact. Relevant endpoints may include inflammatory and immune responses, local tissue responses, hemocompatibility for blood-contacting materials, and degradation-related effects [100,113]. Evaluation should extend beyond microbial endpoints to application-specific measures of tissue organization, integration, regeneration, and function.

## 9. Critical Barriers: Evolution, Selectivity, Biological Realism, and Translation

Strong antimicrobial activity under controlled laboratory conditions does not establish that a biomaterial will remain effective, selective, and clinically useful. Its performance may change when microbial populations adapt, when host or non-target microorganisms are exposed, or when biological, manufacturing, and use conditions differ from those of the original laboratory assessment. The critical question is therefore whether antimicrobial function remains appropriate under the conditions for which the material is intended [67].

### 9.1. Evolutionary Consequences of Antimicrobial Exposure

Experimental evolution of *E. coli* on silver and copper surfaces showed increased tolerance to the antimicrobial surfaces after 30 exposure cycles in some populations. However, this adaptation did not consistently increase the minimum inhibitory or bactericidal concentrations of the corresponding metal ions, and antibiotic cross-resistance was not detected under the tested conditions. This finding shows that evolutionary responses to antimicrobial surfaces can depend strongly on the exposure environment and should be evaluated using conditions relevant to the intended material application [114].

Antimicrobial exposure can impose selection on heterogeneous microbial populations, particularly when susceptible cells are inhibited while less susceptible populations remain capable of growth. For antibiotics, subinhibitory exposure can select for resistance and alter bacterial phenotypes. The outcome depends on the antimicrobial, exposure concentration, duration, and bacterial population [115,116]. Andersson and Hughes specifically review selection and variability under subinhibitory antibiotic exposure [115], while Wistrand-Yuen et al. experimentally demonstrated evolution of high-level resistance during sub-MIC streptomycin exposure in *Salmonella enterica* [116].

Resistance, tolerance, and persistence should be distinguished rather than treated as interchangeable. Resistance enables bacterial growth at antimicrobial concentrations that inhibit susceptible populations and is typically associated with heritable changes. Tolerance permits survival during antimicrobial exposure without necessarily increasing susceptibility thresholds, whereas persistence refers to transient survival of a minority subpopulation that can resume growth after exposure is removed [5].

These distinctions are relevant to antimicrobial biomaterials because a reduction in bacterial viability at a single time point does not establish elimination of surviving populations or predict their response to subsequent exposure. However, evidence from conventional antibiotics should not be assumed to apply equally to all antimicrobial biomaterials. Selection pressures may differ with the antimicrobial mechanism and exposure conditions. Antimicrobial coatings based on metals or AMPs have also been investigated for resistance selection, but evidence from healthcare settings remains limited [117]. Such testing should therefore be interpreted as a risk assessment for the specific material and exposure conditions, rather than as evidence that every antimicrobial material will select for resistance [5,117]. This supports an evolution-aware approach to biomaterial design. The relevant risk should be evaluated according to the antimicrobial mechanism, exposure profile, duration, and microbial population.

### 9.2. Selective Infection Control and Biological Collateral Effects

Three polymer coatings tested against biofilms formed from human saliva reduced bacterial attachment but also changed the composition of the bacterial community. The effects differed among the coatings, showing that an antimicrobial surface can alter a complex microbial community rather than simply reduce the target pathogen. These findings support evaluating effects on relevant non-target microorganisms when the surrounding microbial community contributes to health or disease [118].

Antimicrobial intervention occurs within biological communities rather than against pathogens in isolation. Broad-spectrum antibiotics can suppress commensal microorganisms as well as target pathogens, and disruption of resident microbiota can weaken colonization resistance and permit expansion of opportunistic or resistant organisms [119].

For biomaterials, localization may restrict antimicrobial exposure to the intended site, but it does not by itself establish biological selectivity. The biological effects of an antimicrobial material depend on its mechanism, local concentration, exposure duration, accessibility, and interactions with surrounding tissues and microorganisms. Accordingly, selective antimicrobial materials have been investigated to improve pathogen targeting while limiting effects on host cells and non-target microorganisms, although selectivity remains dependent on the antimicrobial mechanism and application context [67].

The design objective is selective infection control rather than indiscriminate microbial destruction. Evaluation should consider pathogen suppression alongside host–cell compatibility and, where a resident microbial community is relevant to the application, effects on representative commensal microorganisms. No single threshold for acceptable collateral effects applies across all applications; the appropriate balance depends on the anatomical site, duration of exposure, antimicrobial mechanism, and clinical indication [67].

### 9.3. Biological Realism and Strength of Evidence

A three-dimensional wound model incorporated *P. aeruginosa*, *S. aureus*, and *Enterococcus faecalis* into collagen-based scaffolds before implantation into mouse wounds. The model reproduced viable multispecies biofilms and produced measurable local and systemic host responses, including leukocyte infiltration and changes in inflammatory markers. This model illustrates how adding a three-dimensional matrix, multiple bacterial species, and host interaction can reveal biological features that are not captured by simple planktonic assays [120].

The evidentiary value of an antimicrobial result depends partly on the biological features reproduced by the experimental model. Planktonic assays provide controlled conditions for screening and comparison, but they do not reproduce the spatial organization, physiological gradients, or host-associated factors characteristic of established biofilms. Likewise, simplified in vitro biofilm models may not adequately reproduce the environmental conditions encountered in vivo, limiting direct extrapolation to clinical infection [121,122]. Vyas et al. identify the limited resemblance of many conventional in vitro biofilms to those encountered in vivo as a barrier to laboratory-to-clinic translation [121], while Guzmán-Soto et al. emphasize the need to select biofilm models according to the biological context being studied [122].

Increasing model complexity can provide additional biological information, but no universal model progression applies to every biomaterial or infection setting. Depending on the question, evaluation may extend from controlled antimicrobial assays to established biofilms, resistant clinical isolates, biologically conditioned or polymicrobial systems, host-microbe models, dynamic systems, animal studies, and, where justified, clinical investigation. Model selection should reflect the biological feature and claim being tested rather than complexity alone [121,123].

The strength of a biological claim should not exceed the realism of the model supporting it. This principle is particularly important for antibiofilm studies because reductions in biomass, metabolic activity, or viability under a defined laboratory condition do not by themselves demonstrate eradication of an established infection or establish clinical effectiveness. More clinically relevant models can incorporate host-derived factors, tissue characteristics, flow, polymicrobial interactions, and site-specific environmental conditions that influence biofilm physiology and antimicrobial response [121,123]. Kolodkin-Gal et al. emphasize infection-site topography and chemical gradients in model selection [123], while Vyas et al. describe approaches for incorporating host-relevant conditions into biofilm models [121]. 

Accordingly, terms such as “eradication,” “infection control,” and “clinical efficacy” should be used only when the experimental model and endpoints provide evidence appropriate to those claims [37,121]. A reduction in crystal-violet staining in a static biofilm model, for example, should not be interpreted as evidence of clinical eradication of an implant-associated infection.

### 9.4. Translational Barriers from Laboratory Performance to Clinical Use

An optimized 2-aminoimidazole antibiofilm coating for titanium orthopedic implants was tested against a clinical *S. aureus* isolate. The coating retained activity after common sterilization procedures, was applied to a human-scale fracture fixation device, and did not impair fracture healing or osseointegration in a rabbit osteotomy model. This study illustrates that translation requires evaluation of antimicrobial activity together with sterilization stability, application to clinically relevant device geometries, and tissue compatibility [124].

Biological efficacy is only one component of translation. Antimicrobial coatings and other surface-modified biomaterials must retain their intended function while remaining sufficiently stable during processing, sterilization, storage, implantation, and use. Manufacturing, sterilization, long-term stability, protein fouling, safety, and regulatory requirements represent important barriers to translating antimicrobial implant coatings from laboratory development to clinical application [125].

Sterilization is particularly important because processing can alter antimicrobial agents, coatings, or material properties. Evaluation should therefore use the intended finished and processed device, including the applicable sterilization process, rather than assume that results obtained with an unprocessed material will be preserved after manufacturing. Biocompatibility assessment should likewise consider the final finished device and its manufacturing and sterilization processes within the context of its intended use [125,126].

Manufacturing introduces additional requirements for reproducibility and scale. A formulation that performs well under highly controlled laboratory conditions may become less practical when fabrication involves complex processing, multiple active components, narrow process tolerances, or demanding quality control. These considerations are particularly relevant to antimicrobial coatings incorporating active agents such as AMPs, for which characterization, manufacturing consistency, and regulatory requirements can complicate translation from laboratory development to medical-device applications [127].

Long-term safety must be considered alongside antimicrobial efficacy. Changes in coating integrity, degradation, release of active components, or prolonged exposure can alter the biological response over the intended service period. Translation therefore requires evidence that the finished material system retains its intended function while meeting appropriate requirements for safety, durability, reproducibility, and manufacturing quality [125,127]. 

The translational challenge is therefore not simply to reproduce laboratory antimicrobial activity at larger scale, but to demonstrate that the complete system maintains its intended performance under conditions relevant to clinical use. Table 2 summarizes the key domains for evaluating infection-resilient materials, linking each domain to an evidence criterion and the evaluation needed to support the corresponding claim.

## 10. Toward Infection-Resilient Biomaterials: A Coordinated Design Paradigm

The preceding sections show that infection control cannot be reduced to a single material property or antimicrobial mechanism. Microbial establishment, biofilm development, host responses, antimicrobial exposure, tissue recovery, evolutionary pressure, and translational constraints are interconnected. Antimicrobial biomaterials should therefore be designed as interfaces whose functions are selected according to the biological conditions they are intended to encounter. Established approaches include antiadhesive, antimicrobial, antibiofilm, multifunctional, and host-compatible strategies, although these functions are not necessarily integrated within a single system [15,16,67].

This review uses “infection resilience” as an organizing framework for integrating these established functions according to biological stage and therapeutic need. It is presented as a conceptual synthesis rather than as a new classification or established terminology in the biomaterials literature. The central contribution is the integration of antimicrobial, antibiofilm, adaptive, host-compatible, and regenerative functions within a stage- and context-dependent design framework.

### 10.1. Defining Infection Resilience

A mussel-inspired hydrogel coating developed for titanium implants combined an AMP with silicate nanoparticles. The coating was designed to remain attached to the titanium surface while providing antimicrobial activity and supporting osteogenic interactions. In vitro, controlled peptide release reduced biofilm formation, while the coating also showed osteoconductive properties. This system illustrates how several established functions can be incorporated into one material system, while the specific functions remain dependent on the intended biological and clinical context [128].

An infection-resilient biomaterial is defined here as a material system designed to maintain appropriate performance across changing stages of microbial challenge and host response. Depending on the application, this may involve limiting initial colonization, detecting infection-associated changes, activating localized antimicrobial activity, controlling established microbial communities, and facilitating subsequent host recovery. These functions have been explored individually or in combination through antifouling, bactericidal, antibiofilm, responsive, and immunomodulatory strategies [15,67].

The defining feature is therefore not the number of functions incorporated into a material, but their coordination with the biological conditions and therapeutic purpose of the application. A material need not perform every function to be infection-resilient; its functions must instead be appropriate to the microbial state, host environment, intended site, and required duration of performance.

### 10.2. Principles for Designing Infection-Resilient Biomaterials

A titanium implant coating based on titania nanotubes and poly-γ-glutamic acid was designed to provide pH-responsive silver-ion release during implant-associated infection and to support subsequent tissue recovery. In an infected implant model, the coating provided antibacterial activity during the early stage and was associated with a later shift toward a pro-healing macrophage phenotype and improved bone formation. This system illustrates the principle that material functions can be coordinated with different stages of the biological response rather than being activated simultaneously [129].

The evidence reviewed supports several principles for this framework. First, microbial establishment should be limited where feasible. Preventive performance can be evaluated by measuring initial bacterial adhesion and early biofilm development. Antiadhesive and antifouling strategies therefore remain important, provided that microbial exclusion does not compromise the host interactions required at the material interface [15,16].

Second, responsive antimicrobial functions should be evaluated by their signal recognition, activation threshold, and response kinetics. When responsive functions are incorporated, activation should be linked to the conditions for which the system was designed. Stimuli-responsive materials can enable spatial or temporal control of antimicrobial activity, but responsiveness alone does not establish therapeutic value [130].

Third, established microbial communities require endpoints that reflect their biological state. Planktonic susceptibility does not reproduce the structural and physiological characteristics of mature biofilms, and antibiofilm systems may therefore require complementary strategies directed at both biofilm prevention and established communities [8,67].

Fourth, antimicrobial function must remain compatible with host biology. Reduction in microbial burden does not by itself demonstrate appropriate immune regulation, tissue integration, or restoration of function. Evaluation should therefore consider host–cell responses, tissue interactions, and local immune effects alongside antimicrobial performance [15,67].

Fifth, evolutionary and ecological consequences should be considered during design and evaluation. Antimicrobial exposure can impose selection on microbial populations, while intervention can also affect non-target microorganisms. Resistance and tolerance risks and effects on relevant microbial communities should therefore be evaluated alongside antimicrobial efficacy [5,115,119].

Finally, design must account for performance under conditions relevant to clinical use. Laboratory antimicrobial activity alone does not establish durability, manufacturing reproducibility, biological safety, or clinical feasibility. Sterilization, stability, protein fouling, manufacturing, characterization, safety, and regulatory requirements can all constrain translation [125,127]. Evaluation should therefore determine whether the finished material maintains its intended antimicrobial and biological performance under relevant processing and use conditions. This formulation is a synthesis proposed by this review rather than a universal rule established in the literature.

### 10.3. From Multifunctionality to Coordinated Infection Resilience

Multilayered implant coatings have been designed to release different therapeutic agents at different times. One study used degradable multilayers to deliver gentamicin first to control an established biofilm and then release BMP-2 to support bone repair around the implant. In a rat osteomyelitis model, this time-staggered approach combined infection control with subsequent tissue repair. This approach illustrates the distinction between simply adding functions and coordinating their timing according to the biological sequence of infection and recovery [131].

Multifunctionality is an established approach in antimicrobial biomaterials. Materials have been developed to combine antimicrobial and antiadhesive functions, incorporate multiple antimicrobial mechanisms, or integrate antimicrobial activity with other biological functions [16,67]. Multifunctionality, however, should not be equated with infection resilience. A material may provide several capabilities without coordinating when, where, or under which biological conditions they should operate.

The distinction is therefore between functional multiplicity and coordinated performance across changing biological conditions. A material may combine antimicrobial, antibiofilm, responsive, immunomodulatory, or regenerative functions yet remain poorly matched to the microbial state, host response, or intended clinical use. Conversely, an application may require only a limited number of functions when those functions are appropriately selected and coordinated. In this framework, infection resilience is a systems-level design property rather than a measure of functional complexity. Its value depends on the relationship among function, timing, location, biological context, durability, and clinical purpose [16,67].

The conceptual contribution of this review is to organize established antimicrobial, antibiofilm, adaptive, host-compatible, and regenerative functions within a coordinated framework. The proposed framework builds on established approaches in infection-resistant and multifunctional biomaterials, including antiadhesive, bactericidal, antibiofilm, and immunomodulatory strategies, but organizes them around the changing requirements of the host, microbe, and material interface [15,16,67].

**Figure 4:**
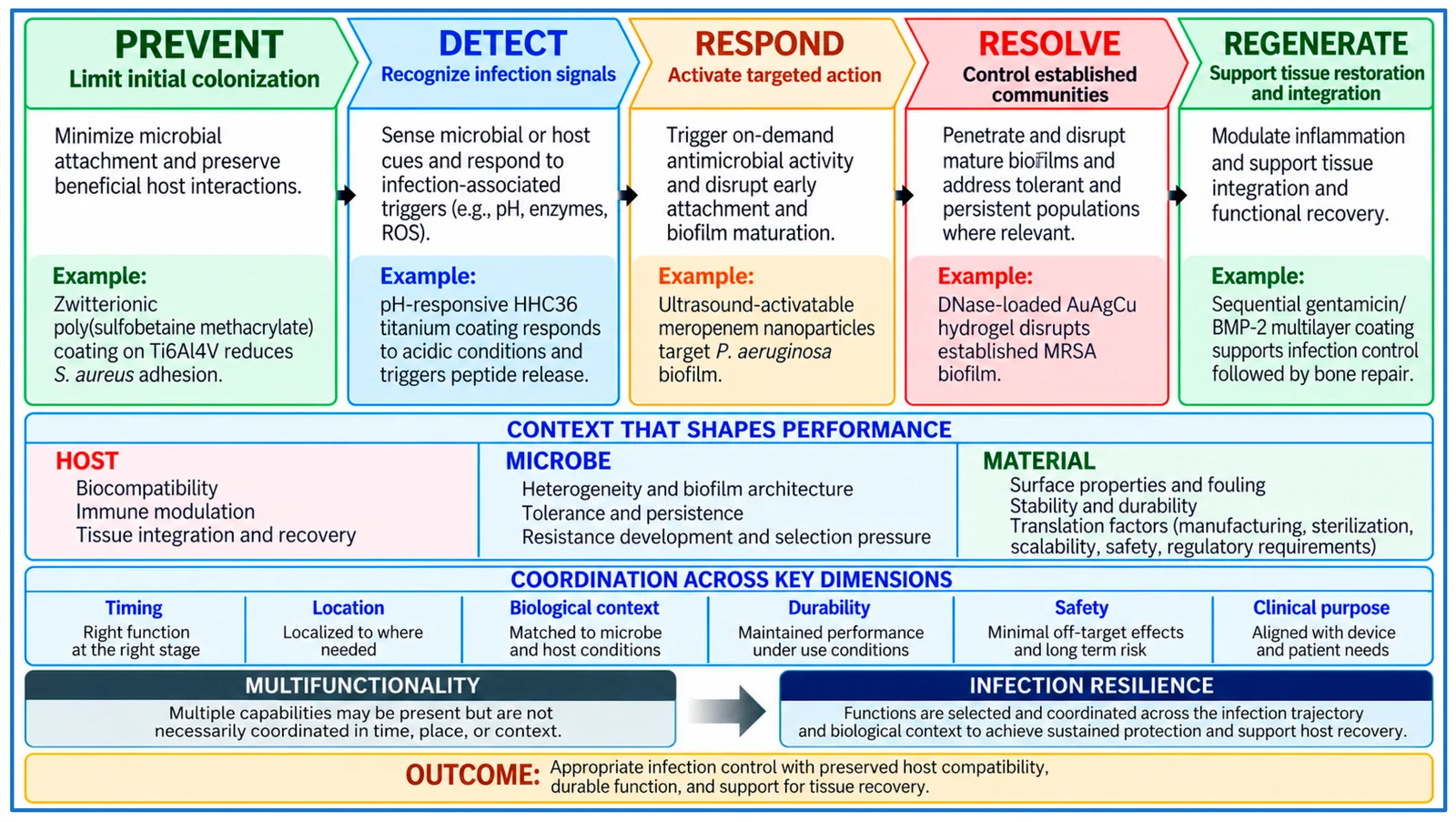
Conceptual framework for infection-resilient biomaterials. The framework coordinates material functions across prevention, detection, antimicrobial response, biofilm control, and tissue recovery according to host, microbial, material, temporal, spatial, safety, durability, and clinical factors. Representative examples illustrate each function, while their inclusion does not imply demonstrated mechanistic synergy.

This formulation is presented as the synthesis proposed by this review, rather than as established terminology or a previously validated design paradigm. Figure 4 summarizes the proposed framework for coordinating material functions as infection and host needs change.

## 11. Conclusions

Antimicrobial biomaterials must be designed in the context of the biological complexity of infection rather than judged by antimicrobial activity alone. Microbial attachment and biofilm development interact with host responses, antimicrobial exposure, microbial adaptation, and changes at the material interface, all of which can affect treatment outcomes. Therefore, this review argues for a shift from multifunctionality as the simple combination of material functions toward coordinated infection resilience in which functions are selected according to the microbial state, host environment, site, and intended duration of use. The framework emphasizes prevention, detection where appropriate, antimicrobial response, support for infection control and resolution, and recovery of the host interface without assuming that every material must perform every function. Ultimately, the goal is not to maximize antimicrobial activity, but to develop biomaterial interfaces that maintain effective infection control while preserving biological compatibility and supporting clinically relevant performance. The infection-resilience framework proposed here is a conceptual synthesis of the current evidence and has not itself been experimentally validated. It should be considered to be a proposed design and evaluation framework rather than an established or validated paradigm. From our perspective, surface-engineered biomaterials that combine durable antiadhesive or antifouling properties with localized antimicrobial or antibiofilm activity represent a promising approach for implant-associated infections. Responsive, immunomodulatory, or regenerative functions may add value when they are appropriate for the intended application. However, no single biomaterial class can currently be recommended for all clinical settings because direct comparative evidence remains limited. The framework brings these requirements together and offers a basis for more biologically relevant evaluation and more purposeful design of next-generation antimicrobial biomaterials.

## Figures and Tables

**Table 1:** Drug-Resistant Pathogens as Biological Stress Tests for Antimicrobial Biomaterials.

Pathogen	Clinical Relevance	Key Biological Challenge	Key Performance Criterion	Refs.
CRAB	WHO critical priority pathogen; important healthcare-associated infection	Carbapenem resistance, biofilm formation, persistence, and isolate variability	Antimicrobial activity is maintained against resistant surface-associated populations	[1,42,44]
MRSA	WHO high-priority pathogen; important implant-associated pathogen	Colonization, biofilm formation, persistence, and host interaction	Bacterial colonization is limited while host compatibility is preserved	[1,2,14]
CRPA	WHO high-priority pathogen; important healthcare-associated and chronic infection challenge	Biofilm-associated tolerance, adaptation, and persistence	Established biofilms are controlled under biologically relevant conditions	[1,5,48]

Abbreviations: CRAB, carbapenem-resistant *Acinetobacter baumannii*; MRSA, methicillin-resistant *Staphylococcus aureus*; CRPA, carbapenem-resistant *Pseudomonas aeruginosa*.

**Table 2:** A Framework for Evaluating Infection-Resilient Biomaterials.

Domain	Evidence Criterion	Representative Evaluation	Refs.
Early microbial establishment	Antimicrobial performance remains effective under relevant microbial conditions.	Initial attachment and early biofilm formation under defined conditions.	[53,58]
Established biofilm	The material reduces or controls an established biofilm.	Mature biofilms; biomass, metabolic activity, viable-cell recovery, and regrowth assessed as distinct endpoints; eradication only when supported by appropriate evidence.	[36,37,121]
Evolutionary effects	Exposure is evaluated for tolerance, adaptation, or resistance under the tested conditions.	Repeated or subinhibitory exposure; susceptibility and survival.	[5,115]
Selective control	Pathogen suppression is achieved while host–cell compatibility and effects on relevant non-target microorganisms are evaluated.	Host–cell compatibility; comparative activity against target and non-target microorganisms.	[67,119]
Biological realism	The model reproduces features relevant to the intended infection.	Host factors, spatial organization, physiological gradients, flow, polymicrobial conditions, and site-specific environments.	[121,122,123]
Durability and manufacturing	The finished material retains its intended function after processing and use.	Sterilization, conditioning, mechanical stability, degradation, reproducibility, and quality control.	[125,127]
Translational evidence	Evidence remains consistent as biological and clinical relevance increases.	Advanced infection models, animal studies, and clinical investigation where justified.	[121,125]

## Data Availability

No new data were created or analyzed in this study.
